# Factors affecting extremity fracture risk in children with attention-deficit/hyperactivity disorder

**DOI:** 10.55730/1300-0144.6047

**Published:** 2025-07-03

**Authors:** Yüksel Sümeyra NARALAN, Esra DEMİREL

**Affiliations:** 1 Department of Child and Adolescent Psychiatry, Faculty of Medicine, Recep Tayyip Erdoğan University, Rize, Turkiye; 2 Department of Orthopaedics and Traumatology, Erzurum Regional Training and Research Hospital, Erzurum, Turkiye

**Keywords:** Attention deficit hyperactivity disorder, extremity fracture, children, methylphenidate, atomoxetine, physical health

## Abstract

**Background/aim:**

Attention-deficit/hyperactivity disorder (ADHD) is increasingly recognized not only for its behavioral and cognitive challenges but also for its potential implications in physical health, particularly injury risk. This study aimed to investigate the incidence of extremity fractures among children and adolescents diagnosed with ADHD, and to evaluate the influence of demographic, clinical, and pharmacological variables—including ADHD subtypes and medication types—on fracture risk.

**Materials and methods:**

This retrospective cross-sectional study included 754 children and adolescents aged 6–18 years old who were diagnosed with ADHD according to the Diagnostic and Statistical Manual of Mental Disorders-5 criteria. Data were collected from electronic health records at a tertiary referral hospital. Variables analyzed included age, sex, ADHD subtype, intelligence quotient (IQ) level, pharmacological treatment status (methylphenidate or atomoxetine), comorbid psychiatric and medical conditions, and fracture history confirmed by clinical and radiological evidence. Binary logistic regression analysis was conducted to identify independent predictors of fracture risk.

**Results:**

The overall incidence of extremity fractures was 15%, with 69% occurring in the upper extremities. Children using ADHD medication had significantly lower fracture rates (9.7%) compared to untreated peers (32.6%, p < 0.001). Logistic regression showed that both methylphenidate (OR = 0.396) and atomoxetine (OR = 0.138) were associated with reduced fracture risk. The inattentive subtype also showed a protective effect. Other factors, such as age, sex, IQ, and comorbidities, were not significantly associated with fracture incidence.

**Conclusions:**

This study highlights a notable reduction in extremity fracture risk among children with ADHD receiving pharmacological treatment, suggesting a possible protective role of stimulant and nonstimulant medications. Subtype-specific risk profiles further emphasize the importance of personalized approaches in ADHD management strategies.

## 1. Introduction

Attention-deficit/hyperactivity disorder (ADHD) is a neurodevelopmental condition that begins in childhood and often continues into adolescence and adulthood, characterized by symptoms of inattention, hyperactivity, and impulsivity [1]. The global prevalence exceeds 5%, with a higher incidence observed in male children [2,3]. Although the diagnosis of ADHD primarily relies on behavioral symptoms, recent studies have shown that individuals with ADHD may be at risk not only in cognitive or academic domains but also in terms of physical health outcomes [4–6].

Notably, in the context of traumatic injuries and accidents, individuals diagnosed with ADHD are reported to have increased physical morbidity due to inattention, impulsivity, and irregular motor behaviors [7–9]. This elevated risk is not limited to superficial injuries; it spans a wide spectrum of serious consequences, including dental, ophthalmological, otorhinolaryngological, and orthopedic traumas [10–13]. Therefore, ADHD should be regarded not only through its psychiatric manifestations but also as a multifaceted clinical condition with systemic implications.

Pharmacological agents commonly used in the treatment of ADHD—particularly methylphenidate and atomoxetine—may reduce the risk of trauma through behavioral regulation [14–18]. However, data also indicate that these medications could potentially result in neutral or adverse outcomes with respect to fracture risk due to their side effects on appetite suppression, growth retardation, and possible alterations in bone metabolism [19–23]. Accordingly, the impact of ADHD pharmacotherapy on physical health outcomes remains a matter of debate.

Most of the studies in the literature address the relationship between ADHD and fracture risk at a general diagnostic level, often without distinguishing between subtypes of ADHD or specific medication types. Furthermore, many of these studies are based on small sample sizes or rely on secondary data sources, limiting the generalizability and clinical applicability of their findings.

This study aims to evaluate the prevalence of extremity fractures among children and adolescents diagnosed with ADHD, compare fracture risks between those receiving pharmacological treatment and those who do not, and analyze the influence of demographic (age, sex) and clinical (cognitive capacity, comorbid conditions) factors on fracture risk. Moreover, this study investigates whether there is differential fracture risks associated with ADHD subtypes and specific pharmacological agents (methylphenidate and atomoxetine). In doing so, it seeks to address a critical gap in the current literature and provide clinically relevant insights for practice.

## 2. Materials and methods

### 2.1. Data collection and annotation

This retrospective cross-sectional study was conducted by reviewing the medical records of children and adolescents diagnosed with ADHD and had follow-up at the Child and Adolescent Psychiatry Outpatient Clinic of Erzurum Regional Training and Research Hospital between January 1, 2019, and December 31, 2020. The inclusion criteria were defined as a diagnosis of ADHD established by a child and adolescent psychiatrist according to DSM-5 criteria, and an age range between 6 and 18 years. Diagnostic procedures included semistructured clinical interviews, parental and teacher versions of the DSM-IV-Based Screening and Assessment Scale for Behavioral Disorders in Children and Adolescents, and, where applicable, the Wechsler Intelligence Scale for Children-Revised Form (WISC-R) and the Wechsler Adult Intelligence Scale-Revised Form (WAIS-R).

All participants included in the study were clinically diagnosed with ADHD according to DSM-5. Based on scores from the DSM-IV-Based Screening and Assessment Scale for Behavioral Disorders in Children and Adolescents, individuals were categorized as follows: those scoring 2 or 3 on at least 6 out of 9 items in the inattention subscale were classified as the inattentive subtype, those scoring 2 or 3 on at least 6 items from the hyperactivity and impulsivity sections were classified as hyperactive subtype, and those meeting the above criteria for both domains were defined as the combined subtype.

Initially, 1140 individuals were assessed. However, those with missing intelligence test data and children under the age of 6, for whom WISC-R could not be administered, were excluded, resulting in a final sample size of 754. The presence of obesity, which could potentially influence bone metabolism, was evaluated using body mass index calculations, and individuals with obesity were excluded from the study. The final sample was assessed for the following variables: sex, age, ADHD subtype, cognitive capacity level, presence of comorbid psychiatric and medical conditions, use and type of pharmacological treatment for ADHD, use of additional psychiatric medication, and history of extremity fracture.

All children and adolescents diagnosed with ADHD were recommended pharmacological treatment. Regarding medication use, participants were divided into two groups: those who received stimulant (methylphenidate) or nonstimulant (atomoxetine) treatment for at least 3 months, and those who were recommended treatment but did not use any medication. Extremity fracture history was verified based on clinical and radiological records available in the hospital information system. All documented fractures occurred during the period in which pharmacological treatment recommendations were active. For medicated participants, documented fractures occurred within the active course of treatment (i.e. after initiation and during the continued use of medication). In addition to treatment subgroups, fracture prevalence by sex was also analyzed comparatively. The study was approved by the Ethics Committee of Erzurum Regional Training and Research Hospital (decision date: April 19, 2021; decision number: 2021/08-156), and all data were analyzed anonymously.

### 2.2. Statistical methods

All analyses were conducted using SPSS Statistics 29.0 (Armonk, NY, USA). The distribution of continuous variables was evaluated using the Kolmogorov-Smirnov test. Descriptive statistics were presented as means, standard deviations, medians, minimum and maximum values, and percentages. The relationships between categorical variables were analyzed using the Pearson chi-squared test, and in cases where expected frequencies were insufficient, the Fisher’s exact test was used.

To identify independent variables potentially associated with extremity fractures, binary logistic regression analysis was performed. A p-value of less than 0.05 was considered statistically significant.

## 3. Results

The study included a total of 754 children, with a mean age of 10.5 years (SD = 2.89) and a median age of 10 years. The participants ranged in age from 6 to 18 years old. Regarding sex distribution, 72.1% (n = 544) were male and 27.9% (n = 210) were female. When examining ADHD subtypes, 59.9% (n = 452) were classified as combined type, 38.6% (n = 291) as inattentive type, and 1.5% (n = 11) as hyperactive type. In terms of cognitive capacity, 79.8% (n = 602) of the individuals were within the normal cognitive range with an intelligence quotient (IQ) ≥ 90, while 20.2% (n = 152) had borderline or low intellectual functioning (IQ < 90) (Table 1).

Among the participants, 76.5% (n = 577) had used pharmacological treatment for ADHD for at least 3 months. Of this group, 43.1% (n = 325) received methylphenidate and 33.4% (n = 252) received atomoxetine. The untreated group consisted of individuals diagnosed with ADHD who had not used any medication. The prevalence of comorbid psychiatric disorders was 42.7% (n = 322), and comorbid medical conditions were present in 24.3% (n = 183). Use of additional psychiatric medication was reported in 15% (n = 113), while 85% (n = 641) reported no such use (Table 1).

Extremity fractures were observed in 15% of the total sample (n = 113), whereas 85% (n = 641) were not observed to have any fractures. Anatomically, 69% of the fractures occurred in the upper extremities, and 31% in the lower extremities. The most frequently affected regions included the distal radius (2.6%), distal humerus (2.4%), distal tibia (1.3%), and metatarsal bones (1.4%) (Table 1).

In the group using ADHD medication, extremity fractures were identified in 9.7% (n = 56), while this rate was significantly higher in the untreated group at 32.6% (n = 57). The difference was statistically significant (p < 0.001). When stratified by sex, fracture prevalence was 37.4% (43/115) in males and 22.6% (14/62) in females, also reaching statistical significance (p = 0.031) (Table 2).

When comparing medication types within the treated group, the fracture rate was 8% (26/325) among those using methylphenidate and 11.9% (30/252) among those using atomoxetine. The difference was statistically significant (p = 0.047) (Table 3).

The overall fit of the logistic regression model was deemed satisfactory (Hosmer–Lemeshow test: χ^2^ = 12.347, p = 0.136). The model correctly classified 85% of the cases, with a Cox and Snell R^2^ of 0.090 and a Nagelkerke R^2^ of 0.159. The analysis showed that individuals with the inattentive subtype of ADHD had a significantly reduced risk of extremity fracture (B = −0.922; p < 0.001; OR = 0.398). Similarly, the use of methylphenidate (B = −0.970; p = 0.002; OR = 0.379) and atomoxetine (B = −2.002; p < 0.001; OR = 0.135) were both significantly associated with a reduction in fracture risk. Other variables, including age, sex, cognitive capacity, comorbid psychiatric or medical conditions, and additional medication use, were not found to be significantly correlated with the outcome variable (p > 0.05) (Table 4, Figure).

## 4. Discussion

This study examined the incidence of extremity fractures and the demographic and clinical factors influencing this risk among children and adolescents aged 6 to 18 years diagnosed with ADHD. Of the 754 participants included, 15% had sustained an extremity fracture, with the majority involving the upper extremities. When exploring the association between ADHD pharmacotherapy and fracture risk, it was observed that both methylphenidate and atomoxetine users had significantly lower fracture rates compared to untreated individuals. Additionally, the inattentive subtype of ADHD was found to exert a protective effect, while other variables such as age, sex, cognitive capacity, and comorbid conditions did not show a statistically significant impact. These findings provide a comprehensive basis for clinical reflection and offer valuable insights for future investigations. Moreover, the results show the potential benefit of pharmacological interventions in mitigating fracture risk in individuals with ADHD.

The primary finding of this study is that children and adolescents diagnosed with ADHD have a markedly elevated risk of extremity fractures, which is significantly reduced in those receiving regular pharmacological treatment. This suggests that ADHD should be understood not only in terms of behavioral and emotional symptoms but also as a multifaceted condition with implications for physical health outcomes.

The fracture incidence of approximately 15% observed in the sample aligns closely with previous reports [24]. However, in a metaanalysis by Seens et al. [25], the fracture rate among individuals with ADHD was reported as 4.83%, with a 2.5-fold increased risk compared to non-ADHD controls. Similarly, a large cohort study conducted by Ziv-Baran et al. [26] found that children with ADHD were 20% more likely to sustain fractures than their non-ADHD peers. While the fracture incidence in this study is similar to that of the general population, it appears higher relative to other ADHD-specific findings. This may be attributed to methodological differences, the exclusion of children under 6 years of age, and/or the inclusion of untreated cases in the current sample.

Furthermore, the observation that 69% of fractures involved the upper extremities is noteworthy and aligns with existing literature. The predominance of distal radius and humerus injuries suggests that fall-related trauma mechanisms may be more prevalent among individuals with ADHD. This reinforces the perspective that the combined effects of inattention, motor coordination difficulties, and impulsivity render these individuals more physically vulnerable.

The fracture rates and anatomical distribution patterns identified in this study are consistent with previous findings. These results highlight the need to consider the physical health consequences of ADHD and to expand psychiatric management strategies to incorporate somatic risks such as injury. In clinical settings, pharmacotherapy should be viewed not only as a tool for symptom control but also as a protective measure against physical trauma.

ADHD is clinically classified into subtypes characterized by distinct behavioral patterns [1]. In this study, the inattentive subtype was associated with a significantly lower incidence of extremity fractures. Logistic regression analysis revealed that individuals with this subtype had approximately a 60% reduction in fracture risk. This finding is notable not only statistically but also clinically, as it directly reflects the impact of behavioral patterns on physical outcomes.

Inattentive ADHD is typically marked by cognitive sluggishness and reduced responsiveness to environmental stimuli, rather than motor hyperactivity or impulsive behavior [6]. The decreased prevalence of risk-taking behaviors and physically hazardous actions in these individuals may account for the lower observed fracture incidence [27]. In contrast, individuals with hyperactivity and impulsivity are known to be more prone to accidents, unregulated reactions to stimuli, and uncoordinated motor actions [8,9].

Despite this differentiation, the effects of ADHD subtypes on physical health outcomes have been rarely explored in the literature. For instance, while the metaanalysis by Seens et al. [25] indicated an overall increased fracture risk, it did not distinguish between ADHD subtypes [13]. Similarly, studies by Ziv-Baran et al. [26] and Chou et al. [28] approached the diagnosis as a single entity without analyzing subtype-specific outcomes. Therefore, the current study contributes uniquely to the literature by addressing this gap through subtype-specific fracture risk assessment.

The observed subtype-specific differences have implications not only for risk assessment but also for treatment planning. Given the lower risk among individuals with the inattentive subtype, intervention strategies might be tailored accordingly. In contrast, those with pronounced hyperactive-impulsive features may require stricter safety protocols and trauma prevention strategies [27]. Recognizing extremity fractures, especially when evaluated from a clinical perspective, is important for individualized treatment planning. Children with the hyperactive-impulsive or combined subtypes may benefit from more proactive fracture prevention strategies, such as increased parental or school supervision, personalized psychoeducation, or participation in structured physical activities that reduce impulsive risk taking. Conversely, children with the inattentive subtype, who appear to be at lower risk for fractures, may not require the same level of environmental modification, allowing clinicians to focus their therapeutic efforts more on cognitive and academic support. Integrating these subtype-based risk profiles into routine practice may improve both safety and overall treatment efficacy in pediatric ADHD management.

Thus, this study shows a significant association between ADHD and fracture risk in addition to ADHD subtypes having distinct physical risk profiles. This distinction should be recognized both in research and clinical practice.

Methylphenidate and atomoxetine, two of the most frequently used pharmacological agents in ADHD treatment, were evaluated in this study for their effects on symptom control and their implications for physical health outcomes. The findings showed significantly lower fracture rates among individuals who used either drug consistently. The fracture incidence was 8% among methylphenidate users and 11.9% among atomoxetine users, compared to 32.6% in those who did not use any medication—an observation that is both statistically and clinically meaningful.

The protective effect of pharmacotherapy on fracture risk has been supported by a growing body of literature. For example, a large-scale Danish study by Dalsgaard et al. [15] reported a significant reduction in traumatic injury risk among medicated children with ADHD. Similarly, Perry et al. [16] found lower fracture rates in individuals treated with methylphenidate, especially with long-term use. A study by Jacob and Kostev [29] in Germany reported a 39% reduction in fracture risk in children receiving pharmacological treatment, with this effect observed for both methylphenidate and atomoxetine.

However, some studies have raised concerns about the potential adverse effects of pharmacotherapy on bone metabolism. Experimental animal studies have suggested that chronic methylphenidate use may impair bone mineral density [20], while certain clinical studies in human samples have proposed that long-term stimulant treatment may negatively affect growth and bone development [21,22]. Nonetheless, the small sample sizes and short follow-up periods of these studies limit their ability to establish causal relationships.

The current findings suggest that the fracture-reducing effect of ADHD pharmacotherapy is mediated by behavioral regulation rather than direct physiological mechanisms. The observed reduction in uncontrolled physical movements, due to better control of impulsivity and hyperactivity, may account for the protective benefit. The similar protective effect seen in both methylphenidate and atomoxetine users further supports the behavioral basis of this mechanism.

The differential fracture risk observed by medication type is also of interest. The lower incidence associated with methylphenidate may reflect sample characteristics, such as the predominance of the combined ADHD subtype and male participants—groups for whom clinicians often prefer methylphenidate due to its rapid efficacy. In contrast, atomoxetine is frequently prescribed in clinical contexts involving comorbid anxiety or learning disorders, or where response to methylphenidate is inadequate. These factors, combined with potential differences in the effects of the drugs on hyperactivity-impulsivity and risk-taking behaviors, may explain the observed variation [14,29]. Future prospective studies that objectively assess impulsivity, hyperactivity, and risk-taking behavior will be instrumental in clarifying these differences.

Overall, the results suggest that pharmacological treatment not only addresses psychiatric symptoms but may also serve as a protective factor against trauma-related outcomes such as extremity fractures. This emphasizes the importance of considering physical safety alongside behavioral management when deciding on treatment strategies.

The finding that 72.1% of the sample were male aligns with literature showing a higher prevalence of ADHD in boys [2,3]. This may be due to the more visible nature of hyperactivity-impulsivity symptoms in males that are more likely to be recognized by families and teachers [3]. Fracture rates were 37.4% in males and 22.6% in females, yet sex was not found to be a significant predictor in multivariate analysis.

Although this result may seem unexpected, the lack of significance may stem from the marked imbalance in sex distribution within the sample. Additionally, the absence of a control group in this study limits the ability to compare sex differences against the general population. While Ziv-Baran et al. [26] identified male sex as a fracture risk factor, and Chen et al. [14] reported similar findings including comorbidities, Prasad et al. [30] also found that 84.6% of fracture cases occurred in males.

Age did not emerge as a significant predictor of fracture risk in this study, despite the inclusion of individuals aged 6–18. In contrast, Chou et al. [28] reported higher fracture incidence in children under 12, with the difference diminishing during adolescence. Guo et al. [31] also indicated that the relationship between age and fracture risk varies by age group rather than following a linear pattern.

The lack of significance for age and sex in multivariate analysis may reflect the confounding effects of other variables such as treatment status, ADHD subtype, and comorbidities. The retrospective design of this study may also have limited the ability to detect such nuanced effects. Future prospective studies involving more homogeneous subgroups and control comparisons may help to clarify these associations.

Other clinical variables such as cognitive capacity, comorbid psychiatric and medical conditions, and additional psychiatric medication use were not significantly associated with fracture risk. The absence of a meaningful difference in fracture rates between those with IQ < 90 and those with normal intelligence suggests that cognitive ability alone may not be a strong determinant of fracture risk. The limited number of studies on this topic, and the tendency to treat cognitive deficits as secondary to behavioral symptoms, may account for the lack of clarity in the literature [32].

No significant associations were observed between fracture risk and comorbid psychiatric diagnoses such as anxiety, depression, or conduct disorder. This suggests that these conditions may not independently determine injury risk, or that they interact complexly with core ADHD symptoms. Duramaz et al. [32] also found that although a history of ADHD was common among children with fractures, comorbid psychiatric diagnoses were not assessed as independent predictors.

Similarly, no significant association was found between fracture risk and medical comorbidities such as asthma, diabetes, or rheumatological conditions. In contrast, Chen et al. [14] suggested that chronic illnesses like asthma might exacerbate fracture risk when cooccurring with ADHD. In the current study, medical conditions were analyzed collectively rather than by specific diagnosis, which may have masked potential subgroup effects.

The use of additional psychiatric medication was also unrelated to fracture risk in this study. This may indicate that such medications (e.g., antidepressants, antipsychotics) do not directly increase fracture risk or may even attenuate it via interactions with core ADHD treatments. Nonetheless, more detailed studies are needed to evaluate pharmacological profiles and interactions.

Although these findings regarding clinical variables diverge from some reports in the literature, the discrepancies may be due to differences in study design, sample size, the absence of a control group, and the retrospective nature of data collection. Importantly, the more behaviorally defined variables—such as ADHD subtype and treatment use—appear to be stronger predictors of fracture risk. These findings offer guidance on which parameters may be most relevant in clinical decision-making. One important limitation of the present study is the absence of a non-ADHD control group. Although fracture rates in untreated ADHD participants offer a partial reference, the lack of direct comparison with healthy peers restricts the generalizability of the findings to broader pediatric populations. This limitation should be acknowledged, especially when interpreting the relative risk conferred by pharmacological treatment or ADHD subtypes. Another limitation of this study is that, despite identifying statistically significant predictors, the explanatory power of the regression model was modest (Nagelkerke R^2^ = 0.159). This suggests that a significant portion of the variance in fracture risk was not explained. It is possible that unmeasured behavioral factors (such as physical activity levels or parental supervision) and environmental influences such as home or school safety conditions also contribute significantly to fracture risk, but this was not captured in the retrospective design. Another limitation pertains to the lack of detailed information regarding the reasons why some participants did not initiate medication despite being recommended. This may introduce a source of selection bias between the treated and untreated groups. For example, families declining pharmacological intervention might differ in terms of socioeconomic status, health literacy, or attitudes toward medical treatment—factors that could independently influence the child’s risk of injury. Such unmeasured differences may compromise internal validity and limit the strength of causal inferences regarding the protective effect of medication. However, to ensure generalizability, large-scale, prospective case-control studies are needed to further explore the factors influencing fracture risk in children with ADHD.

This study showed that children and adolescents diagnosed with ADHD have an increased risk of extremity fractures; however, this risk was significantly reduced among individuals receiving regular pharmacological treatment, particularly with methylphenidate and atomoxetine. The findings suggest that pharmacological intervention may have positive effects not only on behavioral symptoms but also on physical safety.

The protective effect associated with the inattentive subtype highlights the differential roles of ADHD subtypes in physical health outcomes. No significant associations were found between fracture risk and sex, age, cognitive capacity, or comorbid conditions.

Overall, these results emphasize the importance of incorporating considerations related to physical trauma risk into ADHD management. The potential benefit of pharmacotherapy in this regard should be acknowledged, reinforcing that its advantages extend beyond the domain of psychiatric symptom control.

## Figures and Tables

**Figure:**
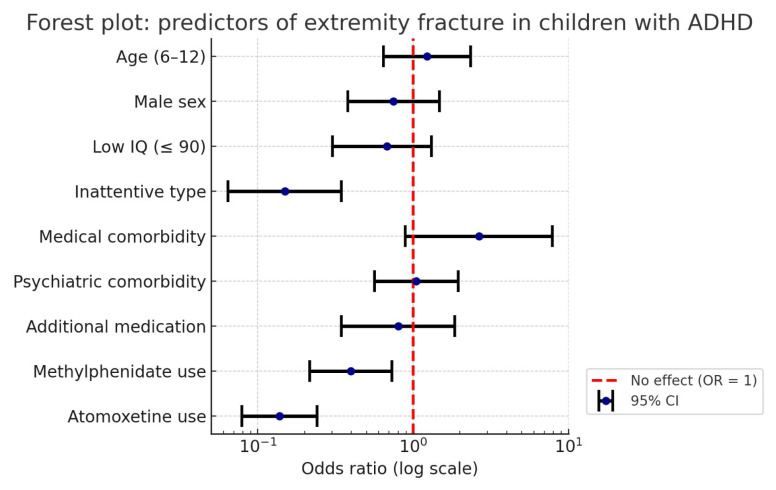
This forest plot illustrates the results of a binary logistic regression analysis examining potential predictors of extremity fracture in children and adolescents diagnosed with ADHD. Odds ratios (OR) and their corresponding 95% confidence intervals (CI) are shown on a logarithmic scale. An OR below 1 indicates a protective effect, while an OR above 1 indicates increased risk. The red dashed line represents the null value (OR = 1). Statistically significant predictors include the inattentive ADHD subtype, and the use of methylphenidate and atomoxetine, all of which are associated with a reduced risk of fracture.

**Table 1:** Demographic and clinical characteristics of participants.

		n	%
**ADHD medication status**	**Medication use (≥3 months)**	577	76.5
	**No medication**	177	23.5
**Comorbid psychiatric disorder**	**Present**	322	42.7
	**Absent**	432	57.3
**Additional psychiatric medication**	**Used**	113	15.0
	**Not used**	641	85.0
**Extremity fracture**	**Present**	113	15
	**Absent**	641	85

Comorbid psychiatric disorders include conditions such as anxiety disorders, depression, and conduct disorder. The incidence of extremity fractures includes diagnoses verified through clinical and radiological records.

**Table 2:** Distribution of extremity fractures by ADHD medication use and sex.

		Extremity fractures		
ADHD medication use		Present	Absent	p
**Used**	**n**	56	521	*<0.001* *
**Not used**	**n**	57	120	
**Sex**		**Present**	**Absent**	p
**Male**	**n**	43	72	*0.031* *
**Female**	**n**	14	48	

* Statistical analysis was performed using the Fisher’s exact test, and p < 0.05 was considered statistically significant.

Fracture status was categorized as either present or absent based on clinical and radiological verification. The “medication use” group includes participants who received regular methylphenidate or atomoxetine treatment for a minimum of 3 months for ADHD. The “no medication” group consists of individuals who were recommended pharmacological treatment but did not initiate any medication.

**Table 3:** Frequency of extremity fractures by type of ADHD medication (methylphenidate vs. atomoxetine).

		Extremity fractures		
ADHD medication type		Present	Absent	p
**Methylphenidate**	**n**	26	299	*0.047* *
**Atomoxetine**	**n**	30	222	

* Statistical significance was determined using the Fisher’s exact test, with p < 0.05 considered the threshold for significance.

Fracture status (present/absent) was confirmed based on radiological and clinical records. The groups were categorized based on the type of ADHD medication used: Methylphenidate: Individuals receiving only stimulant therapy, and Atomoxetine: Individuals receiving only nonstimulant therapy.

**Table 4:** Logistic regression results for factors associated with extremity fractures.

Variable	B	S.E.	Wald	Odds ratio (OR)	95% CI lower	95% CI upper	p
**Age** **6–12 years old**	0.204	0.326	0.391	1.227	0.645	2.335	0.532
**Male sex**	−0.293	0.345	0.722	0.746	0.378	1.472	0.396
**Cognitive capacity IQ<90**	−0.388	0.414	0.877	0.678	0.302	1.309	0.349
**Inattentive ADHD subtype**	−1.902	0.427	19.81	0.149	0.064	0.345	<0.001*
**Comorbid medical condition**	0.974	0.55	3.135	2.648	0.891	7.867	0.077
**Comorbid psychiatric disorder**	0.047	0.313	0.022	1.048	0.565	1.943	0.882
**Use of additional psychiatric meds**	−0.225	0.427	0.277	0.799	0.345	1.853	0.599
**Methylphenidate Use**	−.927	.310	8.957	.396	0.216	0.727	.003*
**Atomoxetine use**	−1.981	.283	49.087	.138	0.079	0.240	<.001*

* Model classification accuracy: 85%. Explained variance: Cox and Snell R^2^ = 0.090, Nagelkerke R^2^ = 0.159 p < 0.05 was considered statistically significant.

Binary logistic regression analysis was conducted with extremity fracture status (present/absent) as the dependent variable. Variables included in the model: age group (reference: 6–12 years), sex, IQ level (IQ < 90), ADHD subtype (reference: hyperactive/combined), presence of comorbid medical and psychiatric conditions, use of additional psychiatric medication, and ADHD medication type. Odds Ratios (OR) indicate the likelihood of fracture for each variable. Values <1 suggest reduced risk; values >1 suggest increased risk. Model fit was assessed using the Hosmer–Lemeshow test, which showed acceptable fit (p = 0.136).

Methylphenidate and atomoxetine effects are calculated relative to the reference group (children not using ADHD medication).
